# Validation of a Dish-Based Semiquantitative Food Questionnaire in Rural Bangladesh

**DOI:** 10.3390/nu9010049

**Published:** 2017-01-10

**Authors:** Pi-I. D. Lin, Sabri Bromage, Md. Golam Mostofa, Joseph Allen, Emily Oken, Molly L. Kile, David C. Christiani

**Affiliations:** 1 Department of Environmental Health, Harvard T.H. Chan School of Public Health, Boston, MA 02115, USA; pil864@mail.harvard.edu (P.-I.D.L.); jgallen@hsph.harvard.edu (J.A.); molly.kile@oregonstate.edu (M.L.K.); 2 Research Center of Environmental Medicine, Kaohsiung Medical University, Kaohsiung 807, Taiwan; 3 Department of Nutrition, Harvard T.H. Chan School of Public Health, Boston, MA 02115, USA; sbromage@mail.harvard.edu; 4 Department of Environmental Research, Dhaka Community Hospital, Dhaka 1217, Bangladesh; mostofa07@gmail.com; 5 Department of Population Medicine, Harvard Medical School and Harvard Pilgrim Health Care Institute, Boston, MA 02215, USA; emily_oken@harvardpilgrim.org

**Keywords:** food frequency questionnaire, food diary, duplicate food sample, validation study, adult male equivalent, Bangladesh

## Abstract

A locally validated tool was needed to evaluate long-term dietary intake in rural Bangladesh. We assessed the validity of a 42-item dish-based semi-quantitative food frequency questionnaire (FFQ) using two 3-day food diaries (FDs). We selected a random subset of 47 families (190 participants) from a longitudinal arsenic biomonitoring study in Bangladesh to administer the FFQ. Two 3-day FDs were completed by the female head of the households and we used an adult male equivalent method to estimate the FD for the other participants. Food and nutrient intakes measured by FFQ and FD were compared using Pearson’s and Spearman’s correlation, paired *t*-test, percent difference, cross-classification, weighted Kappa, and Bland–Altman analysis. Results showed good validity for total energy intake (paired *t*-test, *p* < 0.05; percent difference <10%), with no presence of proportional bias (Bland–Altman correlation, *p* > 0.05). After energy-adjustment and de-attenuation for within-person variation, macronutrient intakes had excellent correlations ranging from 0.55 to 0.70. Validity for micronutrients was mixed. High intraclass correlation coefficients (ICCs) were found for most nutrients between the two seasons, except vitamin A. This dish-based FFQ provided adequate validity to assess and rank long-term dietary intake in rural Bangladesh for most food groups and nutrients, and should be useful for studying dietary-disease relationships.

## 1. Introduction

Understanding dietary habits is important for studying the development and progression of chronic illnesses. Accurate assessment of dietary habits is crucial in large, prospective epidemiological studies to provide a holistic understanding of health status. Having reliable dietary data allows researchers to examine the relationship of variations in food and nutrient intakes with the susceptibility to adverse effects from environmental factors and diseases. In Bangladesh, limited dietary intake data is available in most epidemiological studies; as a result, most studies had not accounted for individual variations in dietary intakes. Many national surveys employed a labor-intensive 24-h recall method to collect data at the household level [1,2,3,4,5,6,7], but individual habitual dietary data is lacking. Although household data are useful for designing nutrition interventions, more expensive and long-term dietary surveillance is needed to study and prevent chronic diseases.

The food frequency questionnaire (FFQ), which is comprised of a predefined list of food and beverages with response categories to indicate consumption frequency, is a low-cost tool and has been the most widely used method for ranking participants’ dietary and nutrient intake in epidemiologic studies [8]. The validity of a FFQ in measuring relative dietary intake is important. Only one FFQ in Bangladesh using a food-based approach had previously been validated [9]. In rural Bangladesh, where typical dietary patterns are characterized by various mixed food dishes rather than single food ingredients, using this food-based FFQ created problems of respondent fatigue and poorer quality of reported information [10,11,12]. In addition, the previously validated FFQ used food composition tables from the US and Indian nutrient databases to calculate nutrient values, which likely did not reflect the most accurate nutrient values of locally produced food, as food composition varies from country to country depending on the species of plants and animals, agricultural technology, climactic condition, processing, and storage circumstances [10].

In this study, we employed a dish-based semi-quantitative FFQ previously developed for a longitudinal study investigating arsenic exposure and biomarker responses in Bangladesh [13,14] and used the 2013 Food Composition Table in Bangladesh to calculate nutrient intakes [15]. We assessed the validity of the dish-based FFQ by comparing the intakes calculated using the FFQ with intakes measured by two 3-day weighed food diaries (FDs). FD has been acknowledged as an appropriate reference for FFQ validation [10,16] since FD does not share common measurement errors with the FFQ, such as recall bias and errors from the conception of serving size and interpretation of questions [10]. The examination of the validity employed a multifaceted approach, utilizing six statistical tests, including Spearman’s rank-order correlation, paired *t*-test, percent difference, cross-classification, weighted Kappa coefficient, and Bland–Altman analysis [17].

## 2. Materials and Methods

### 2.1. Participant Selection

We invited 248 subjects, from 47 households, concurrently enrolled in a longitudinal arsenic biomonitoring study in rural Pabna, Bangladesh, from 2001–2005 [13,14] to participate in the FFQ study. The recruitment process has been previously described [18]. One hundred and ninety subjects agreed to participate and provided written informed consents (Figure S1). All subjects completed one individual FFQ and the female head of the household in charge of food preparation completed two 3-day FDs. The sample size of 190 had greater than 95% power to detect matched pair mean difference in intake of any food or nutrient larger than 0.27 (α = 0.05, two-tails). The study was approved by the Human Subjects Committees of the Dhaka Community Hospital and the Harvard T.H. Chan School of Public Health (P10565/0204 MOLE, approved 8 November 2001).

### 2.2. FFQ

A dish-based semi-quantitative FFQ was previously developed to assess subjects’ habitual dietary intake over the past 12 months. Development was based on a prior questionnaire used in a national-level nutritional survey conducted in Bangladesh and followed recommended guidelines on FFQ development [10,16]. Through focus group discussion with local community residents and dieticians from the Dhaka Community Hospital, the FFQ was constructed using commonly consumed dishes in Bangladesh from five major categories, including cereal and bread-based dishes; vegetable-based dishes; legume and pulse-based dishes; fish, poultry, meat, and egg-based dishes; and milk-based dishes. The dishes that contributed to each of the categories are listed in Table S1. Supplement and fluid consumption was also assessed. The final FFQ consisted of 42 dishes which were accepted based on the frequency of consumption and considered to make a substantial contribution to nutrient intakes of rural Bangladeshi adults. The FFQ assessed quantity, portion size, and frequency for each item consumed. Five frequency options were used: ‘daily’, ‘weekly’, ‘monthly’, ‘yearly’, and ‘never’. The pre-specified portion sizes included large plate, medium plate, small plate, large bowl, medium bowl, small bowl, glass, cup, large spoon, small spoon, and piece. Visual aids for portion sizes were provided using locally used plates, bowls, cups, and serving utensils. Trained interviewers guided participants to ensure complete responses. All subjects completed one individual FFQ. The FFQ was administered in January 2004, at least one month before the collection of the FD.

### 2.3. Weighed FD

We instructed the female head of each household to keep a written FD at the time of consumption with quantities and portion sizes of all foods and beverages consumed at the time of consumption for three consecutive days in both winter (February–March 2004) and summer (June–August 2004), with one weekend day included in each period. We instructed the subject to report portion sizes using the same portion size option as specified in the FFQ, including large plate, medium plate, small plate, large bowl, medium bowl, small bow, glass, cup, large spoon, small spoon, and piece. We also asked subjects if the diet recorded each day reflected their usual diet or not. Concurrent with the FD collection, the female heads of the households provided a duplicate food sample (DFS) of each portion consumed and was reimbursed with $9 USD compensation after each sampling period (summer and winter). One field team member visited each participant at midday and in the evening to weigh DFS collected from morning to midday and from midday to evening, respectively. Detailed methods on the process of DFS have been described previously [13,14]. Measured food weights were used to calculate portion sizes for food dishes recorded in the FD of the female head of the household, whereas food weights for other family members were estimated by multiplying the consumption of all foods from the female head of the household by each other family member’s respective adult male equivalent (AME). The AME is an expression of the relative estimated consumption of foods, based on the relation between an individual’s predicted caloric requirement and those of an adult male [19]. Adult male equivalents (AMEs) have previously been calculated for different age and sex groups in Bangladesh using data from the 2010 Household Income and Expenditure Survey (HIES) [20], following the methodology of Bermudez et al. [21].

### 2.4. Analysis of Food and Nutrient Intake

Two trained technicians double-entered the primary data from FFQs and FDs to ensure accuracy. Daily nutrient intakes were calculated based on the 2013 Food Composition Table for Bangladesh [15]. The Food Composition Table includes nutrients of 328 food items and nutrient compositions of 27 single ingredient and 11 multi-ingredient recipes. For dishes in the FFQ that were not available in the Food Composition Table, their nutrient compositions were calculated based on nutrition values of the cooked ingredients according to average weighed recipes provided by local dietitians at DCH using the nutrient retention factors (RF) and yield factors (YF) provided in the 2013 Bangladesh Food composition Table. The mixed recipe calculation method was used, specifically, the YF is applied at the recipe level and RF was applied at the ingredient level [22]. Weighed recipes for food dishes were collected from 20 households in the study area and average recipes were calculated per 100 g for each food dish in different seasons. Average daily food intake assessed by FFQ was calculated using the formula:

(1) $${\mathrm{Intake}}_{FFQ_{i}}=\sum^{}\left(q_{ij}\times f_{ij}\times p_{j}\right)$$

where *q* is the reported quantity, *f* is the reported frequency per day, *p* is the pre-specified portion size in grams, for subject *i* and dish *j*. Average daily food intake assessed by FD was calculated using the formula:

(2) $${\mathrm{Intake}}_{FD_{i}}=\frac{\sum^{}\left(q_{j}\times mp_{j}\right)}{n}\times \;\frac{AME_{i}}{AME_{k}}$$

where *q* is the recorded quantity in the FD, *mp* is the measured food weight in gram for dish *j*, *AME* is the adult male equivalent consumption for household members in Bangladesh based on age and sex [20] (previously calculated for different age and sex groups of the Bangladeshi population using methodology of Bermudez et al. [21]), *n* is the number of days the FD was collected, and *k* is the person who filled out the FD in the household to which *i* subjects belonged. Food group intake was calculated by directly adding the consumption of each dish by food category, e.g., ‘grain, cereal, and bread-based dish’, ‘vegetable-based dish’, etc. Nutrient intakes and energy intake were calculated by multiplying the nutrient and energy values per 100 g of food adjusted for inedible waste from the Food Composition Table for Bangladesh [15]. The contribution from each food dish was then added for each participant to create the total nutrient and energy intakes.

### 2.5. Statistical Analysis

Demographic characteristics of the study population were tabulated. Food and nutrient intakes not normally distributed, as indicated by the Shapiro–Wilk test, were logarithmically transformed using the formula log(x + 1) to achieve a normal distribution. Body mass index (BMI) was calculated for participants aged 20 years of age or older using the formula weight (kg)/height squared (m^2^). For participants under 20 years of age, we calculated age- and sex-specific BMI Z-scores using the WHO Growth Reference Charts [23]. FFQ responses with three missing components (missing quantity, missing frequency, and missing portion size) were imputed with the null value (no consumption). Responses with one or two missing components were imputed with the sample median conditioning on the values of the non-missing, e.g., a response with reported quantity of ‘2’, frequency of ‘daily’, and missing portion size was imputed with the median portion size of samples reporting quantity of ‘2’ and frequency of ‘daily’.

Seasonal variations were tested by the Shrout–Fleiss intraclass correlation coefficient (ICC) between the winter and summer average dietary intakes derived from the two 3-day FD [24]. The ICC, which is defined as a ratio of between-person variance to total variance, ranges from 0–1 and can be an indicator of reproducibility of dietary intake between the two seasons. Values from 0–0.25 indicate poor reproducibility; 0.25–0.4 indicate low reproducibility; 0.4–0.6 indicate fair reproducibility; 0.6–0.75 indicate good reproducibility; and 0.75–1.0 indicate excellent reproducibility [25,26].

To quantify the accuracy of the two 3-day FD in capturing individual’s intake level, the unobservable (hypothetical) correlation coefficient (*r_h_*) between observed and true mean nutrient intakes of the population during the period of observation was calculated using the following formula [27]:

(3) $$r_{h}=\sqrt{\frac{D}{D+\left(S_{w}^{2}/S_{b}^{2}\right)}}\;$$

where *S_w_* is the observed within-subject variance, *S_b_* is the observed between-subject variance, and *D* is the number of days of FD collected for each participant.

The validity of the FFQ was assessed by comparing the intake of each nutrient/food group estimated from the FFQ with that estimated from the average of the two 3-day FD. Means and standard deviation (SD) of intakes for each nutrient and food group, as obtained from the FFQ and the FD, were computed separately, and means were compared using paired *t*-tests. Differences, ratios, and percent differences between mean values obtained with the FFQ and the FD were calculated to assess the level of agreement and direction of error [28,29]. Pearson’s product moment correlation and Spearman’s rank-order correlation were calculated to measure the strength and direction of the association. Since total energy intake can introduce extraneous variation in recorded food intake, intake estimates were adjusted for total energy intake using the residual method [30]. Briefly, residuals, which were derived from the model’s food or nutrient intakes regression on total energy intake, were standardized to the predicted log-transformed dietary intake of a subject with the average total energy intake of all subjects in the study. Energy-adjusted correlations were de-attenuated for within-person variability in measurements, which tends to reduce the correlation coefficient toward zero [31]. The deattenuation was performed using the formula:

(4) $$R=\sqrt{r(1+\lambda_{x}/n_{x})}$$

in which *λ_x_* represents the ratio of the within- and between-person variances for *x*, and *n_x_* represents the number of replicates for the *x* variable [10]. For this study, *n* = 3. Within- and between-person variations were calculated by one-way ANOVA for food and nutrient intakes estimated by the two 3-day FD.

In addition to using Spearman’s correlation, FFQ’s ability to rank participants correctly was assessed by cross-classification of energy-adjusted nutrient intakes into quintiles. Subjects that were assigned by the FFQ to the opposed extreme quintile of intakes based on their responses in the FD were considered grossly misclassified. The percentage of agreement indicated subjects with intakes ranked in the same quintile, though the agreement may occur by chance [32]. To assess the agreement accounting for chance, we used the weighted kappa statistic (κ_w_) with Cicchetti–Allison weights, which measured the inter-rater agreement of categorical items accounting for natural ordering of categories [33,34,35].

Bland–Altman plots with differences between the measurements (*y*-axis) against the mean of the two measures (*x*-axis) for each subject were analyzed to assess the presence of proportional bias, as well as the direction of the bias between the FFQ and two 3-day FD for all nutrients and food intakes [32,36,37,38]. We also calculated the Bland–Altman Spearman correlation coefficient between the mean of the two methods and the mean difference of the two methods to indicate the association between the size of the error, the presence of proportional bias, and the direction thereof.

We performed a sensitivity analysis restricted to only the FDs from the 47 female heads of household to assess the validity of the FFQ using Pearson and Spearman correlation. The correlations were also adjusted for energy intake using the residual method and the deattenuated correlations were calculated accounting for within-person variability in measurements.

Results were considered statistically significant at a two-tailed α level of 0.05. Statistical analysis was performed by using SAS software (release 9.4; SAS Institute Inc., Cary, NC, USA).

### 2.6. Integrative Interpretation of Statistical Outcomes

Multiple statistical tests were implemented to provide comprehensive evaluations on the various facets of validity. For the ease of visualization, results for the six statistical tests were color-coded based on levels of validity using criteria proposed by reviews of validity on dietary assessment methods [17]. Specifically, a correlation coefficient ≥0.40 was considered good, 0.20–0.39 was considered acceptable, and <0.20 was considered poor. Paired *t*-test of the mean intake was considered good when *p* > 0.05 and poor if *p* ≤ 0.05. Percentage difference of the mean intake of 0.0%–10.9% was considered good, 11.0%–20.0% was considered acceptable, and >20.0% was considered poor. Cross-classification with ≤10% in opposite quintile was considered good and >10% was considered poor.

Weighted Kappa statistics of ≥0.61 was consider good, 0.15–0.59 was consider acceptable, and <0.15 was considered poor. A Bland–Altman correlation coefficient with a *p*-value > 0.05 was considered good, whereas *p*-value ≤ 0.05 was considered poor.

## 3. Results

All of the 190 subjects had one FFQ and two 3-day FDs. None of the FDs were collected on days of unusual diet (e.g., religious holiday). Table 1 presents the socio-demographic and lifestyle characteristics of the study subjects. Females comprised 54.21% of the study subjects. The cohort averaged 31.5 ± 14.2 years of age and a median BMI of 19.5 kg/m^2^. Most of the participants had education levels lower than primary education. Housewives comprised 40.53% of the participants, 16.32% were students, 8.42% were factory labors, and 8.42% were businessmen. There was no missing data for the sex, age, BMI, or education variables. Fourteen percent of the job type category was missing values. Quantity values were missing in 0.06%, frequency values were missing in 0.37%, and portion size values were missing in 0.78% of the FFQ responses, before imputation.

### 3.1. Seasonal Variability

Table 2 presents the ICCs between food and nutrient intakes of the two 3-day FD. ICC for daily average energy intake was high (0.80, *p* < 0.001), suggesting similar energy intake between different seasons. Intakes of beverages (ICC = 0.82, *p* < 0.001) and grain, cereal, and bread-based dishes (ICC = 0.78, *p* < 0.001) had the least seasonal variation. Intake of fruits (ICC = 0.24, *p* < 0.001) and legume- and pulse-based dishes (ICC = 0.20, *p* < 0.001) had large seasonal variations. Macronutrient intakes had small seasonal variations. Intake of vitamin A had the highest seasonal variation among all nutrient intakes (ICC = 0.38, *p* < 0.001).

### 3.2. Correlation of FD with True Intake

The unobservable (hypothetical) correlation coefficient (*r_h_*) between intakes observed from the two 3-day FD and true mean nutrient intakes of the population during the period was 0.928. With *r_h_* >0.90, 80% of the individuals can be correctly classified into thirds of the distribution and less than 1% are grossly misclassified with 90% confidence [39]. The correlation between macro- and micronutrient intakes from the two 3-day FD and true mean nutrient intake ranged from 0.411 for zinc to 0.881 for protein (Table 2).

### 3.3. Validity

Table 3 presents the comparison of the average daily food group intakes assessed by FFQ and FD. Daily average consumption of grains, cereal, bread-based dishes, fish, poultry, meat, egg-based dish, milk-based dishes, and beverages were overestimated by the FFQ compared to the FD, whereas the FFQ underestimated consumptions of vegetable-based dishes and fruits. Pearson’s and Spearman’s rank correlation yielded similar correlation estimates, we only presented the results of the Pearson’s correlation. The unadjusted Pearson’s correlation coefficient ranges from 0.16 for vegetable-based dishes to 0.75 for fruits. After adjusting for total energy, Pearson’s correlation coefficients increased for all food groups, except grain, cereal, bread-based dishes, and milk-based dishes. The deattenuated correlation corrected for within-person variation in the FD increased the correlation estimates of all food categories, especially for legume- and pulse-based dishes, indicating greater within-person variations as compared with between-person variations in the consumption of food in this category. Accounting for the effect of attenuation, the coefficients ranges from 0.25 for vegetable-based dishes to 0.90 for milk-based dishes.

Table 4 presents the average daily nutrient intakes and correlations between values derived from the FFQ and the FD. The trend of overestimation and underestimation of nutrient intakes was consistent with that of food categories. For instance, consistent with the overestimation of grain, cereal, and bread-based dishes, FFQ overestimated the carbohydrates available by 56% (FFQ:FD = 1.56); on the other hand, the underestimation of fruit intake concurred with lower L-ascorbic acid intake estimated by FFQ (FFQ:FD = 0.78). The unadjusted Pearson’s correlations for nutrient intakes ranged from 0.08 for total cryptoxanthin to 0.38 for fat and iron. Energy adjustments improved the correlations for most nutrients, especially for carbohydrates (0.25–0.54), fiber (0.34–0.45), and protein (0.36–0.51), which agreed with the energy-adjusted improvement in Pearson’s coefficients found on vegetable based dishes (0.16–0.21) and legumes, pulses, and seed-based dishes (0.23–0.34). The energy-adjusted correlation coefficients for food or nutrient intakes between the FFQ and FD were all statistically significant (*p* < 0.001).

Analysis of Bland–Altman plots revealed that most macronutrient and micronutrient intakes did not show significant proportional bias and most of the points fell within the 95% limits of agreement (Figure S2), and no statistically significant association was observed between the differences and means for those nutrient intakes (Bland–Altman Spearman’s correlation *p*-value > 0.05). Outliers were found for intakes of iron, sodium, zinc, vitamin A, and vitamin B6 (Bland–Altman plots not shown). However, Bland–Altman Spearman’s correlations for these nutrient intakes were not statistically significant both before and after the removal of the outliers, suggesting no significant bias was present. The intake of carbohydrate, calcium, potassium, alpha-carotene, total cryptoxanthin, niacin equivalents, and L-ascorbic acid exhibited linear treads on Bland–Altman plots, among which all had a positive proportional bias except for carbohydrates, which had negative proportional bias, i.e., as the mean of carbohydrate intake measured by the two methods increases, the mean difference decreases.

The sensitivity analysis using only the two 3-day FD from the 47 female heads of the household and found similar results comparing to using the AME method. The overall analysis on the subset of 47 women did show stronger corrections for most intakes but, qualitatively, the results were similar. Data with only the 47 female heads of household are presented in Tables S2–S4.

### 3.4. Classification

Results of the cross-classification of food and nutrient intakes estimated by the FFQ and the FD are shown in Table 5. A high proportion of study participants (>70%) was correctly categorized into the same or adjacent quintile for estimates of all food groups and nutrient intakes. Intakes of fish, poultry, meat, egg-based dishes and legumes, pulses, and seed-based dishes had low proportions of grossly misclassified subjects (2.96% and 4.24%, respectively), whereas the intakes of beverages had a high proportion (11.21%). The weighted kappa coefficient matched the cross-classification finding, showing highest weighted kappa for fish, poultry, meat, and egg-based dishes (0.41, 95% CI: 0.35–0.46), and the lowest for beverages (0.07, 95%: 0.00–0.14). All nutrient intakes had a small proportion (average of 6.2%) of being grossly misclassified, among which total dietary fiber (0.90% grossly misclassified, weighted kappa 0.42, 95% CI: 0.33–0.47) was the lowest. Calcium, copper, phosphorus, L-ascorbic acid, beta-carotene, sodium, retinol, vitamin B6, and vitamin A had higher proportions of being grossly misclassified (>0.5%). However, none of the nutrient intakes had more than 10% in the opposite quintile, suggesting nutrient intakes estimated from FFQ and FD had a good agreement at the individual level [17].

### 3.5. Integrative Interpretation of Statistical Outcomes

Table 6 presents the interpretation and summary of six statistical tests comparing average daily nutrient intakes calculated by FFQ and FD. Summary results showed that total energy intake measured by the FFQ had no evidence of statistically significant proportional bias (*p* = 0.04) and that the FFQ agreed well with the FD in classifying total energy intake, although this agreement may be partly due to chance. FFQ had good validity to capture macronutrient intakes (protein, fat, fiber) at the group level, except for carbohydrates, which had a large percent difference and presence of bias with a significant negative Bland–Altman Spearman’s correlation, i.e., the difference became more negative as the mean of the consumption increases. The validity for mineral intakes (calcium, iron, magnesium, phosphorus, potassium, sodium, zinc, and copper) was good at the group level, except for calcium and potassium, which had the presence of proportional bias in the positive direction, i.e., as average intake increases, so does the difference of intake. The supporting individual level validity was acceptable for mineral intakes. Micronutrient intakes had good group and individual level validity for thiamin, riboflavin, niacin, and folate, with no presence of bias. Intake of L-ascorbic acid measured by FFQ had poor validity at both the group and individual levels. Most nutrient intakes measured by FFQ had poor complete agreement with intake measured by FD, suggesting that the FFQ is not suitable for measuring the absolute intakes.

## 4. Discussion

We developed a dish-based semi-quantitative FFQ consisting of 42 food items to assess long-term dietary habits of participants in a longitudinal arsenic biomonitoring study in rural Bangladesh. Performance of the FFQ was evaluated by comparing average food intake and nutrient intakes derived from this instrument with those recorded in two 3-day FD using a combination of six statistical tests. Total energy intake measured by the two 3-day FD had good-to-strong correlations (*r* > 0.7), and the same was true for all macronutrient intakes, dietary fiber, and some micronutrients, including potassium, retinol, vitamin D, niacin, vitamin B6, and folate, after accounting for within-person variations. Caution must be exercised when using the FFQ to assess populations with wide ranges of carbohydrate and potassium intakes since proportional bias existed at the group level. The validity of the FFQ in assessing intake of vitamin A, L-asorbic acid, and total cryptoxanthin was questionable, indicated by weak association, a low level of agreement, and the presences of bias between the FFQ and the two 3-day FD. The FFQ was also not suitable to measure absolute intake.

Compared with previously validated FFQ in Bangladesh that used the USDA nutrient database [9], our FFQ had stronger deattenuated correlation for total energy, protein, carbohydrate, dietary fiber, potassium, iron, magnesium, thiamin, copper, zinc, calcium, folate, retinol, vitamin C, and vitamin E. The use of the Food Composition Table in Bangladesh also allowed us to assess the intake of additional micronutrients that were not available in the previous FFQ, including alpha-carotene, beta-carotene, total cryptoxanthin, and vitamin D. We did not observe the problem of under-reporting of meat and over-reporting of fruits that was observed in the previous food-based FFQ used in Bangladesh [9] and other Westernized countries [40,41,42,43]. The level of overestimation and underestimation of food consumption measured by the FFQ, as compared to those recorded in the FD, was also much lower. Contrastingly, our FFQ overestimated the consumption of grain, cereal, and bread-based dishes, which were not observed in the previous food-based FFQ in Bangladesh. Our dish-based FFQ included a dish of homemade snacks, of which the main ingredient is flour. Consumption of homemade snacks may be considered a symbol of better economic status in rural Bangladesh; thus, its consumption may be over-reported. Since the previous food-based FFQ did not included snacks, it did not observe this problem.

The seasonal variation in nutrient intake of our study population during the study period was small for total energy, protein, and carbohydrate intakes. Larger seasonal variations were found for vitamin D, beta-carotene, and vitamin A intakes. Small seasonal variation for total energy was expected since energy intake and expenditure is sensitivity-regulated according to body size and metabolism level. Protein and carbohydrates are the main contributors to daily energy intake and are distributed across different types of foods and thus expected to have small seasonal variations. Micronutrients, on the contrary, tend to concentrate in specific foods, so the level of micronutrient intakes is highly dependent on food choices and food availability [10]. Previous studies in Bangladesh showed evidence of striking seasonal variations in vitamin A consumption [44,45,46], and a similar observation was found based on our result of the FD.

In settings with wide seasonal variation in diet, the ability to assess the validity of a FFQ in measuring long-term intakes of micronutrients is usually limited and the number of days required for dietary monitoring is greater, in order to allow for true estimation of intakes [47]. A previous study had found poor validity associated with food and nutrient intakes that had large seasonal variations [44]. The utilization of the dish-based approach in our study reduced the severity of this problem. Instead of asking the consumption of a long list of fruits and vegetables, whose availability varied largely with seasons, our strategy was to ask the consumption of vegetable- and fruit-based dishes in the FFQ, i.e., leafy vegetables (sak), fried vegetable (bhaji), mixed vegetables (labra), mashed vegetables (bhorta), vegetable curry (torkarir jhole), dal with vegetables, meat with grains, legumes, vegetables (dhansak), fruit, mashed fruit (bhorta), and fruit pickle (aachar). By asking about the consumption of food dishes, we reduced the variability in consumption levels caused by the availability of certain food ingredients. We are also confident that the average of the mixed dish recipes collected in different seasons from 20 households was representative of the nutrient composition for the food dishes.

Our study has several other strengths. All of our FFQs were obtained by trained interviews via in-person, face-to-face interviews. Interviewers can help collect more detailed data; they encourage participation, and can reduce missing data on the dietary assessment. Only 0.4% of all FFQ questions had a missing response. Compared to self-administered FFQ, where food items may be left blank for various reasons, i.e., because the food was not consumed, because of difficulties remembering the frequency or amount of intake, or due to an oversight [48], having interviewers enabled us to clarify the reason for missing and provided more accurate assessment of dietary intake. This advantage is particularly important in areas, such as Bangladesh, where rural people are not closely familiar with epidemiological studies and have low literacy levels, where having an interviewer is crucial and important. All of the dishes recorded in the FD were available in the FFQ, suggested our FFQ had good ability in capturing the range of food consumed by the study population.

The main limitation of the study was that the FD was only collected from the female head of the household who was in charge of meal preparation, but not for all participants. In Bangladesh, it is common for the female head of the households to be in charge of cooking meals for the entire family at home and for all family members to eat meals cooked at home. Therefore, we found that it was appropriate to estimate the FD for the other members of the household using the AME fraction. Successfully implementation of the AME depends on accurate estimate of energy requirement and ensuring that the consumption of all food is proportional to one’s energy requirement. We used energy requirements derived from a national household survey in Bangladesh, which carefully calculated caloric requirements based on body weight, basal metabolic rate, and physical activity levels for sex and age groups [20]. Although the AME method had not worked consistently well across nations, studies had found good applicability in Bangladesh [49,50]. Sensitivity analysis among the 47 female heads of household supported our use of the AME method, in that it increased the sample size and gave higher statistical power to provide disaggregated results for different age and sex groups.

Another limitation was that the number of FD days collected in the study was only six days due to limited resources and manpower. The lack of sufficient days of FD collection might weaken the statistical power to capture the dietary variation of some micronutrients with high variance ratios [51]. Nevertheless, our study found that the dietary data captured by the two 3-day FD did have higher than 80% power to accurately classify the population into tertiles with 95% confidence for total energy, protein, fat, carbohydrate, folate, niacin, riboflavin, iron, thiamin, potassium, phosphorus, and vitamin E [52].

## 5. Conclusions

The dish-based semi-quantitative FFQ developed to assess long-term habitual diet for rural Bangladeshi population provided valid estimates on relative intakes of food dishes and nutrients both at the group and the individual levels for total energy, all macronutrients, and some micronutrients, such as folate, retinol, thiamin, and riboflavin. The instrument had enough statistical power to accurately rank and classify dietary intake into at least three levels. The dish-based FFQ had low participant burden and generated data that was easy to analyze, and is a useful tool to assess dietary intake in large epidemiology studies.

## Figures and Tables

**Table 1:** Socio-demographic characteristics of study participants.

Variables *		*n*	%
Sex (*n* = 190)			
	Male	87	45.79
	Female	103	54.21
Age (years) (*n* = 190)			
	<10	5	2.6
	11–20	53	27.9
	21–30	41	21.6
	31–40	45	23.7
	41–50	29	15.3
	51–60	10	5.3
	>60	7	3.7
	Median	30.0	
	Mean	31.3	
	SD	14.7	
Job Type (*n* = 190)			
	Farmer	2	1.0
	Agricultural labor	1	0.5
	Factory labor	16	8.4
	Businessman	16	8.4
	Craftsman	0	0.0
	Office worker	4	2.1
Housewife		77	40.5
	Student	31	16.3
	Jobless	7	3.7
	Others	10	5.3
	missing	26	13.7
BMI Z-score (for age 5–19 years, *n* = 48)			
	1 SD	5	10.4
	0 SD	5	10.4
	−1 SD	12	25.0
	−2 SD	13	27.1
	−3 SD	7	14.6
	−4 SD	6	12.5
BMI (kg/m^2^, for age >20 years, *n* = 142)			
	<18.1	30	21.1
	18.1–20.5	39	27.5
	>20.5	73	51.4
Median		20.8	
	Mean	20.9	
	SD	3.6	
Education (for age >20 years, *n* = 142)			
	Illiterate	25	17.6
	Able to write	50	35.2
	Primary education	24	16.9
	Secondary education	21	14.8
	Higher secondary education	14	9.9
	College/graduate	4	2.8
	Post-graduate education	4	2.8
	Missing	0	

* Children aged 5–19 years were included in the age, sex, and job type values, but were not included in the BMI and education values; children’s BMI values were separately presented as BMI Z-score using the WHO Growth Reference Charts [23]. BMI = body mass index (kg/m^2^); SD = Standard Deviation.

**Table 2:** Correlation of average daily food and nutrient intakes obtained from the two 3-day food diaries (FD) by the female head of the household, and the unobservable (hypothetical) correlation coefficient (*r_h_*) between observed intake (from FD) and true mean nutrient intakes of the population during the study period (*n* = 47).

Dietary Intake ^1^		ICC ^2^	*p*-Value	*r_h_*
**Energy (kcal)**		0.80	<0.001	0.928
**Food Group**				
	Beverages	0.82	<0.001	0.942
	Grain, Cereal, Bread based	0.78	<0.001	0.931
	Milk based	0.53	<0.001	0.820
	Vegetable based	0.53	0.66	0.825
	Fish, Poultry, Meat, Egg based	0.50	<0.001	0.821
	Fruits	0.24	<0.001	0.672
	Legumes, Pulses, Seeds based	0.20	<0.001	0.616
**Nutrients**				
	Alpha-carotene (mcg)	0.85	<0.001	0.748
	Potassium (mg)	0.80	<0.001	0.844
	Niacin, preformed (mg)	0.80	<0.001	0.767
	Protein (g)	0.80	<0.001	0.881
	Carbohydrate available (g)	0.79	0.02	0.851
	Thiamin (mg)	0.78	<0.001	0.862
	Phosphorus (mg)	0.78	<0.001	0.837
	Riboflavin (mg)	0.76	<0.001	0.869
	Ash (g)	0.74	<0.001	0.654
	Calcium (mg)	0.73	<0.001	0.759
	Vitamin B6 (mg)	0.73	<0.001	0.779
	Total cryptoxanthin (mcg)	0.72	<0.001	0.712
	Zinc (mg)	0.71	<0.001	0.411
	Niacin equivalents (mg)	0.70	<0.001	0.834
	Beta-carotene equivalents (mcg)	0.70	<0.001	0.748
	Copper (mg)	0.70	<0.001	0.795
	Niacin equivalents from tryptophan (mg)	0.69	<0.001	0.870
	Folate (mcg)	0.69	<0.001	0.848
	Total dietary fiber (g)	0.69	<0.001	0.643
	Magnesium (mg)	0.68	<0.001	0.547
	Vitamin E (mg)	0.66	<0.001	0.806
	L-ascorbic acid (mg)	0.62	<0.001	0.768
	Fat (g)	0.60	<0.001	0.813
	Sodium (mg)	0.59	<0.001	0.729
	Retinol (mcg)	0.53	0.19	0.751
	Iron (mg)	0.50	<0.001	0.852
	Vitamin D (mcg)	0.49	<0.001	0.787
	Beta-carotene (mcg)	0.45	<0.001	0.709
	Vitamin A (mcg)	0.38	<0.001	0.534

^1^ Food intakes and nutrient intakes listed in the descending order of the intraclass correlation coefficient (ICC); ^2^ Calculated based on log-transformed intake values.

**Table 3:** Degree of association and level of agreement between average daily food group intakes by FD and FFQ.

Food Group	Average Daily Consumption (Serving/Day)					Level of Agreement			Correlation Coefficient *		
	FD		FFQ			FFQ-FD ^1^		FFQ:FD ^2^	Unadjusted ^3^	Energy-Adjusted ^4^	Deattenuated ^5^
	Mean	SD	Mean	SD	*p*-Value ^6^	Mean	SD		Pearson	Pearson	Pearson
Grain, Cereal, Bread	4.70	1.67	9.83	2.42	<0.001	5.14	2.30	2.22	0.42	0.33	0.35
Vegetable	1.88	1.13	1.53	0.25	<0.001	−0.38	1.05	0.91	0.16	0.21	0.25
Legumes, Pulses, Seeds	1.15	0.24	1.16	0.14	0.620	0.00	0.26	1.03	0.23	0.34	0.55
Fish, Poultry, Meat, Egg	1.30	0.30	1.74	0.89	<0.001	0.50	0.85	1.41	0.41	0.42	0.51
Milk	1.02	0.11	1.29	0.36	<0.001	0.23	0.29	1.21	0.68	0.66	0.80
Fruits	2.08	2.94	1.66	0.89	0.060	−0.49	2.28	1.13	0.75	0.79	≠
Beverages	1.08	0.28	1.60	1.19	<0.001	0.55	1.05	1.47	0.65	0.85	0.90

* For all correlations, *p* < 0.001. ^1^ FFQ-FQ represents the difference between intake measured by FFQ and intakes measured by FD; ^2^ FFQ:FD represents the percent ratio of paired FFQ and FD intake measurements; ^3^ Calculated based on log-transformed value of average daily consumptions; ^4^ Calculated based on log-transformed value of average daily consumptions with adjustment for total energy intake using the residual method; ^5^ Correction were calculated based on energy-adjusted correlation accounting for random within-individual error in the two 3-day FD; ^6^ Paired *t*-test comparing FFQ and FD in daily average intakes. ≠ Correction coefficient not calculated due to the very large ratio of within-person to between-person variances.

**Table 4:** Degree of association and level of agreement between average daily nutrient intakes by food diary (FD) and by semi-quantitative food questionnaire (FFQ).

Nutrients (Unit)	Average Daily Intake (Unit/Day)					Level of Agreement			Correlation Coefficient between FD and FFQ *		
	FD		FFQ			FFQ-FD ^1^		FFQ:FD ^2^	Unadjusted ^3^	Energy-Adjusted ^4^	De-attenuated ^5^
	Mean	SD	Mean	SD	*p*-Value ^6^	Mean	SD		Pearson	Pearson	Pearson
Protein (g)	44.1	14.6	50.8	13.1	<0.001	6.8	15.5	1.26	0.36	0.51	0.58
Fat (g)	23.0	12.5	21.3	8.7	0.125	−1.7	13.3	1.12	0.38	0.45	0.55
Carbohydrate available (g)	214.6	49.8	326.7	107.8	<0.001	112.1	106.2	1.56	0.25	0.54	0.63
Total dietary fiber (g)	23.7	8.4	26.4	7.6	0.001	2.7	9.6	1.20	0.34	0.45	0.70
Ash (g)	9.2	3.0	10.4	3.0	<0.001	1.2	3.9	1.23	0.19	0.20	0.31
Calcium (mg)	345.5	160.2	299.8	99.1	<0.001	−45.7	167.8	0.98	0.30	0.26	0.34
Iron (mg)	16.1	5.4	21.0	16.7	<0.001	4.9	16.2	1.36	0.38	0.40	0.47
Magnesium (mg)	297.1	92.2	334.8	102.3	<0.001	37.7	117.6	1.20	0.32	0.37	0.68
Phosphorus (mg)	655.1	226.0	873.7	268.9	<0.001	218.7	313.6	1.46	0.30	0.33	0.39
Potassium (mg)	1346.9	463.5	1312.9	386.9	0.438	−33.9	520.5	1.07	0.32	0.45	0.53
Sodium (mg)	939.9	581.9	1033.6	785.4	0.187	93.7	939.1	1.54	0.13	0.17	0.23
Zinc (mg)	6.9	2.3	9.4	3.0	<0.001	2.5	3.3	1.46	0.30	0.36	0.87
Copper (mg)	2.5	0.7	2.7	0.5	0.002	0.2	0.7	1.14	0.21	0.23	0.29
Vitamin A (mcg)	192.0	138.6	334.2	171.1	<0.001	142.2	193.4	2.39	0.21	0.27	0.51
Retinol (mcg)	36.1	45.0	43.5	30.3	0.017	7.4	49.9	3.96	0.17	0.28	0.37
Beta-carotene equivalents (mcg)	2586.6	2594.6	3591.8	1552.5	<0.001	1005.2	2823.7	3.31	0.15	0.20	0.27
Alpha-carotene (mcg)	835.4	656.4	660.3	288.8	<0.001	−175.1	695.0	1.85	0.13	0.22	0.29
Beta-carotene (mcg)	1855.1	1562.4	2972.3	1544.2	<0.001	1117.2	1922.3	2.29	0.17	0.21	0.30
Total cryptoxanthin (mcg)	552.0	400.5	404.5	185.6	<0.001	−147.5	428.2	1.30	0.08	0.22	0.31
Vitamin D (mcg)	2.2	1.2	1.9	0.6	0.002	−0.3	1.2	1.09	0.23	0.45	0.57
Vitamin E (mg)	2.9	1.3	3.3	0.7	<0.001	0.4	1.3	1.32	0.18	0.21	0.26
Thiamin (mg)	1.4	0.3	1.6	0.2	<0.001	0.3	0.3	1.22	0.24	0.33	0.38
Riboflavin (mg)	1.2	0.2	1.4	0.2	<0.001	0.2	0.2	1.22	0.24	0.33	0.38
Niacin equivalents (mg)	6.4	3.3	6.3	3.1	0.761	−0.1	4.0	1.26	0.28	0.30	0.36
Niacin, preformed (mg)	7.8	2.4	11.3	2.6	<0.001	3.5	3.2	1.53	0.26	0.25	0.33
Niacin equivalents from tryptophan (mg)	6.0	3.0	7.3	1.9	<0.001	1.3	3.0	1.48	0.31	0.36	0.41
Vitamin B6 (mg)	1.6	0.4	1.9	0.3	<0.001	0.3	0.4	1.25	0.30	0.38	0.49
Folate (mcg)	140.2	53.1	140.1	40.5	0.984	−0.1	58.4	1.10	0.27	0.26	0.31
L-ascorbic acid (mg)	121.7	68.6	66.4	28.4	<0.001	−55.3	71.1	0.78	0.14	0.14	0.18

* For all correlations, *p* < 0.001. ^1^ FFQ-FQ represents the difference between intake measured by food frequency questionnaire (FFQ) and intakes measured by FD; ^2^ FFQ:FD represents the percent ratio of paired FFQ and FD intake measurements; ^3^ Calculated based on log-transformed value of average daily consumptions; ^4^ Calculated based on log-transformed value of average daily consumptions with adjustment for total energy intake using the residual method; ^5^ Correction were calculated based on energy-adjusted correlation accounting for random within-individual error in the two 3-day FD; ^6^ Paired *t*-test comparing FFQ and FD in daily average intakes.

**Table 5:** Agreement of cross-classification quintiles for food intake assessed by food diary (FD) and by semi-quantitative food questionnaire (FFQ).

Intakes	Same Quintile (%)	Adjacent Quintile (%)	One Quintile Apart (%)	Extreme Quintile (%)	Weighted Kappa (95% CI)
Food Group					
Grain, Cereal, Bread based	26.75	42.00	24.34	6.90	0.20 (0.14, 0.26)
Vegetable based	27.11	37.29	27.23	8.37	0.14 (0.08, 0.20)
Legumes, Pulses, Seeds based	24.40	46.28	25.10	4.24	0.21 (0.15, 0.27)
Fish, Poultry, Meat, Egg based	35.27	46.60	15.17	2.96	0.41 (0.35, 0.46)
Milk based	35.38	30.84	24.95	8.84	0.21 (0.11, 0.30)
Fruits	37.35	32.52	23.80	6.33	0.30 (0.21, 0.40)
Beverages	28.51	36.69	23.60	11.21	0.07 (0.00, 0.14)
Nutrient Intake					
Protein (g)	32.15	45.53	18.89	3.43	0.34 (0.28, 0.41)
Fat (g)	38.77	40.69	18.75	1.80	0.43 (0.38, 0.49)
Carbohydrate available (g)	42.83	33.45	19.48	4.23	0.40 (0.33, 0.47)
Total dietary fiber (g)	33.89	44.63	20.58	0.90	0.42 (0.37, 0.47)
Ash (g)	30.25	37.93	25.32	6.47	0.19 (0.13, 0.25)
Calcium (mg)	29.24	38.46	24.09	8.20	0.14 (0.08, 0.20)
Iron (mg)	27.84	42.85	23.04	6.26	0.22 (0.16, 0.28)
Magnesium (mg)	27.96	42.11	22.78	7.13	0.20 (0.14, 0.26)
Phosphorus (mg)	27.12	38.18	26.12	8.58	0.12 (0.06, 0.18)
Potassium (mg)	28.62	44.39	23.60	3.39	0.29 (0.23, 0.34)
Sodium (mg)	30.42	40.01	20.60	8.97	0.17 (0.10, 0.23)
Zinc (mg)	30.40	39.58	23.76	6.28	0.20 (0.14, 0.27)
Copper (mg)	26.40	40.03	25.07	8.50	0.11 (0.04, 0.17)
Vitamin A (mcg)	29.62	33.46	27.18	9.74	0.09 (0.03, 0.15)
Retinol (mcg)	28.39	38.18	24.46	8.97	0.13 (0.06, 0.20)
Beta-carotene equivalents (mcg)	27.82	39.76	24.43	8.00	0.10 (0.03, 0.16)
Alpha-carotene (mcg)	27.38	44.04	24.87	3.70	0.19 (0.12, 0.25)
Beta-carotene (mcg)	27.32	38.36	25.56	8.76	0.09 (0.02, 0.15)
Total Cryptoxanthin (mcg)	24.30	45.61	23.83	6.27	0.15 (0.09, 0.21)
Vitamin D (mcg)	35.66	38.28	22.42	3.64	0.32 (0.27, 0.37)
Vitamin E (mg)	25.16	40.04	27.03	7.76	0.10 (0.03, 0.16)
Thiamin (mg)	30.94	45.62	21.09	2.34	0.32 (0.26, 0.38)
Riboflavin (mg)	35.24	38.85	21.79	4.11	0.30 (0.24, 0.36)
Niacin equivalents (mg)	23.78	43.50	25.61	7.11	0.13 (0.07, 0.19)
Niacin, preformed (mg)	33.68	38.66	23.46	4.19	0.30 (0.23, 0.36)
Niacin equivalents from tryptophan (mg)	28.63	39.81	25.18	6.40	0.17 (0.11, 0.23)
Vitamin B6 (mg)	26.55	40.23	23.51	9.73	0.10 (0.04, 0.16)
Folate (mcg)	31.57	38.17	23.76	6.49	0.19 (0.13, 0.26)
L-ascorbic acid (mg)	30.96	38.91	21.54	8.58	0.08 (0.04, 0.11)

**Table 6:** Statistical test outcomes and interpretations ^1^ for nutrient intake.

Validation Type	Association	Test of Agreement				Presence, Direction and Extent of Bias
		Complete Agreement	Size and Direction of Error	Including Chances	Excluding Chances	
Statistical Method	Spearman’s Correlation ^2^	Paired *t*-Test (*p*-Value)	Percent Difference ^3^ (%)	Cross-Classification (% in Opposite Quintiles)	Weighted Kappa	Bland–Altman Spearman Correlation (*p*-Value)
Intake (unit/day)						
Energy (kcal)	0.35 ^^^	<0.001	44.0%	9.30	0.14	−0.16 (0.040)
Protein (g)	0.46	<0.001	15.3%	3.43	0.34	0.21 (0.073)
Fat (g)	0.45	<0.001	−7.2%	1.80	0.43	0.24 (0.052)
Carbohydrate available (g)	0.50	<0.001	52.2%	4.23	0.40	−0.32 (<0.001)
Total dietary fiber (g)	0.43	<0.001	11.5%	0.90	0.42	0.08 (0.332)
Ash (g)	0.22	<0.001	12.6%	6.47	0.19	0.04 (0.615)
Calcium (mg)	0.24	<0.001	13.2%	8.20	0.14	0.46 (<0.001)
Iron (mg)	0.32	<0.001	30.3%	6.26	0.22	−0.07 (0.408)
Magnesium (mg)	0.30	<0.001	12.7%	7.13	0.20	0.17 (0.071)
Phosphorus (mg)	0.30	<0.001	33.4%	8.58	0.12	0.01 (0.913)
Potassium (mg)	0.41	0.168	−2.5%	3.39	0.29	0.31 (<0.001)
Sodium (mg)	0.17	<0.001	10.0%	8.97	0.17	−0.15 (0.071)
Zinc (mg)	0.37	<0.001	35.9%	6.28	0.20	0.10 (0.214)
Copper (mg)	0.20	<0.001	8.1%	8.50	0.11	0.15 (0.065)
Vitamin A (mcg)	0.24	<0.001	74.1%	9.74	0.09	0.09 (0.261)
Retinol (mcg)	0.26	0.686	20.5%	8.97	0.13	0.07 (0.374)
Beta–carotene equivalents (mcg)	0.19	<0.001	38.9%	8.00	0.10	0.26 (0.081)
Alpha-carotene (mcg)	0.19	<0.001	−21.0%	3.70	0.19	0.41 (<0.001)
Beta-carotene (mcg)	0.15	<0.001	60.2%	8.76	0.09	0.22 (0.001)
Total Cryptoxanthin (mcg)	0.21	<0.001	−26.7%	6.27	0.15	0.36 (<0.001)
Vitamin D (mcg)	0.41	<0.001	−13.5%	3.64	0.32	0.52 (0.293)
Vitamin E (mg)	0.21	<0.001	15.1%	7.76	0.10	0.32 (0.310)
Thiamin (mg)	0.28	<0.001	18.4%	2.34	0.32	0.08 (0.293)
Riboflavin (mg)	0.24	<0.001	19.0%	4.11	0.30	0.29 (0.002)
Niacin equivalents (mg)	0.22	0.463	−0.8%	7.11	0.13	0.44 (<0.001)
Niacin, preformed (mg)	0.25	<0.001	44.2%	4.19	0.30	−0.15 (0.051)
Niacin equivalents from tryptophan (mg)	0.24	<0.001	22.3%	6.40	0.17	0.29 (0.082)
Vitamin B6 (mg)	0.30	<0.001	20.9%	9.73	0.10	0.20 (0.051)
Folate (mcg)	0.25	0.613	−0.1%	6.49	0.19	0.15 (0.063)
L-ascorbic acid (mg)	0.17	<0.001	–45.4%	8.58	0.08	0.66 (<0.001)

^1^ Interpretation criteria for statistical tests (good in green, acceptable in yellow, poor in red); ^2^ Energy adjusted Spearman’s correlation between average daily nutrient intakes calculated from FFQ and FD (all *p*-values < 0.001); ^3^ (FFQ-FD)/FD × 100%. ^^^ Unadjusted Spearman’s correlation between average daily energy intakes calculated from FFQ and FD (*p*-values < 0.001).

## Supplementary Materials

The following are available online at http://www.mdpi.com/2072-6643/9/1/49/s1, Figure S1: Flowchart of the study participants, Figure S2: Bland–Altman plots for selected nutrient intakes showing agreement between paired means and differences in nutrient intakes measured by FFQ and FD. Table S1: Food dishes in each of food categories in the semiquantitative dish-based Food Frequency Questionnaire (FFQ), Table S2: Socio-demographic characteristics of the 47 female heads of household, Table S3: Degree of association and level of agreement between average daily food group intakes by FD and FFQ reported by the 47 female heads of households, Table S4: Degree of association and level of agreement between average daily nutrients intakes by FD and by FFQ reported by the 47 female heads of the household.
