# Supplementary material for: Validation of a Dish-Based Semiquantitative Food Questionnaire in Rural Bangladesh
Source: Nutrients. 2017 Jan 10;9(1):49. doi: 10.3390/nu9010049 (PMC5295093; doi:10.3390/nu9010049)
Supplement: Supplementary file 1 [file nutrients-09-00049-s001.docx]

Supplementary Materials: Validation of a Dish-Based Semiquantitative Food Questionnaire in Rural Bangladesh

Pi-I. D. Lin-Mruk, Sabri Bromage, Md. Golam Mostofa, Joseph Allen, Emily Oken, Molly L. Kile and David C. Christiani

50 families randomly selected from a longitudinal As biomarker monitor study

Eligible participants

(47 families, *n = 248*)

3 families declined to participate in the duplicate food sample collection

Final participants in the validation study

(*n = 190*)

*N = 58* did not fill out the FFQ

**Figure S1.** Flowchart of the study participants. FFQ: Food Frequency Questionnaire.

| 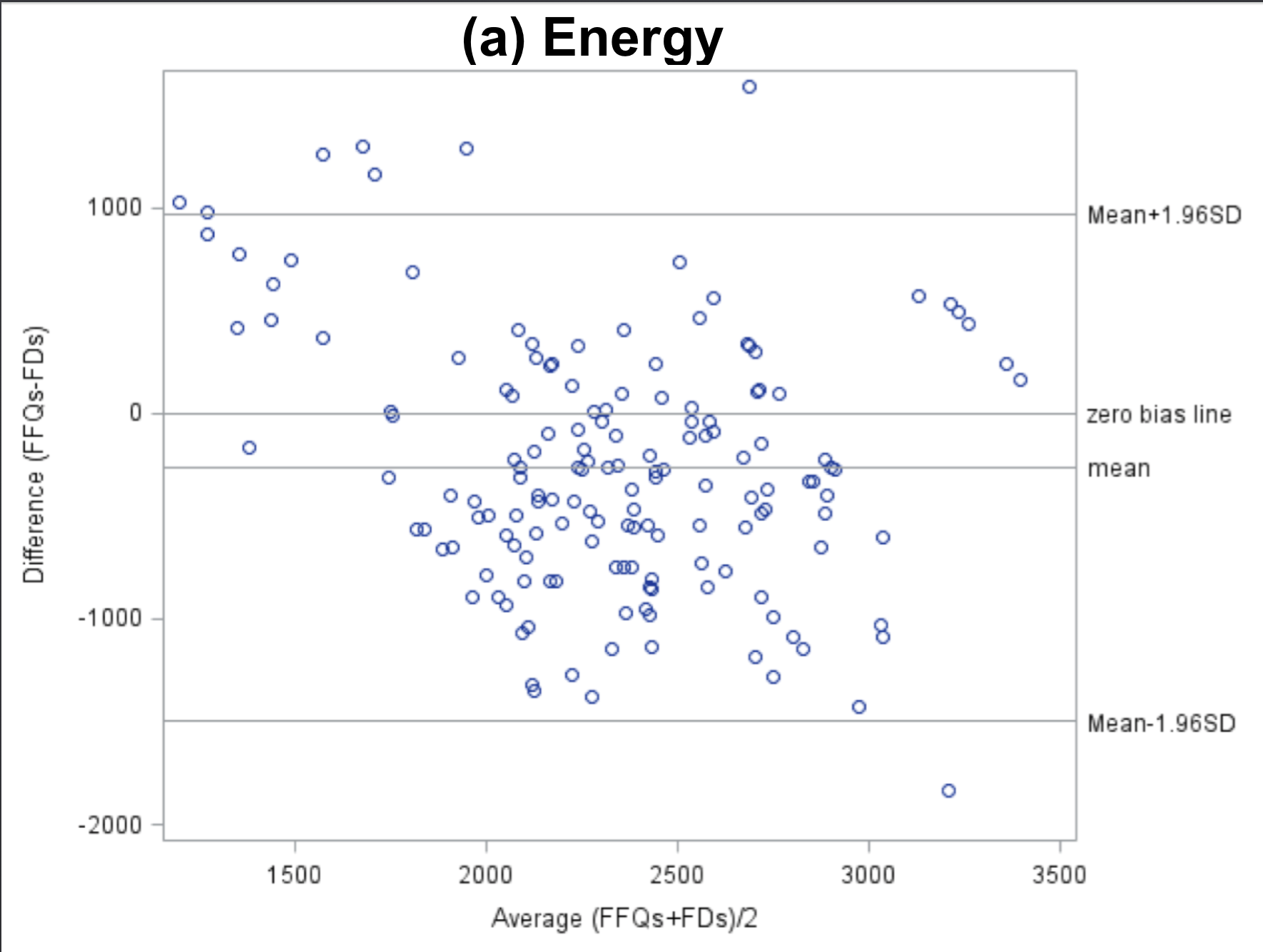 | 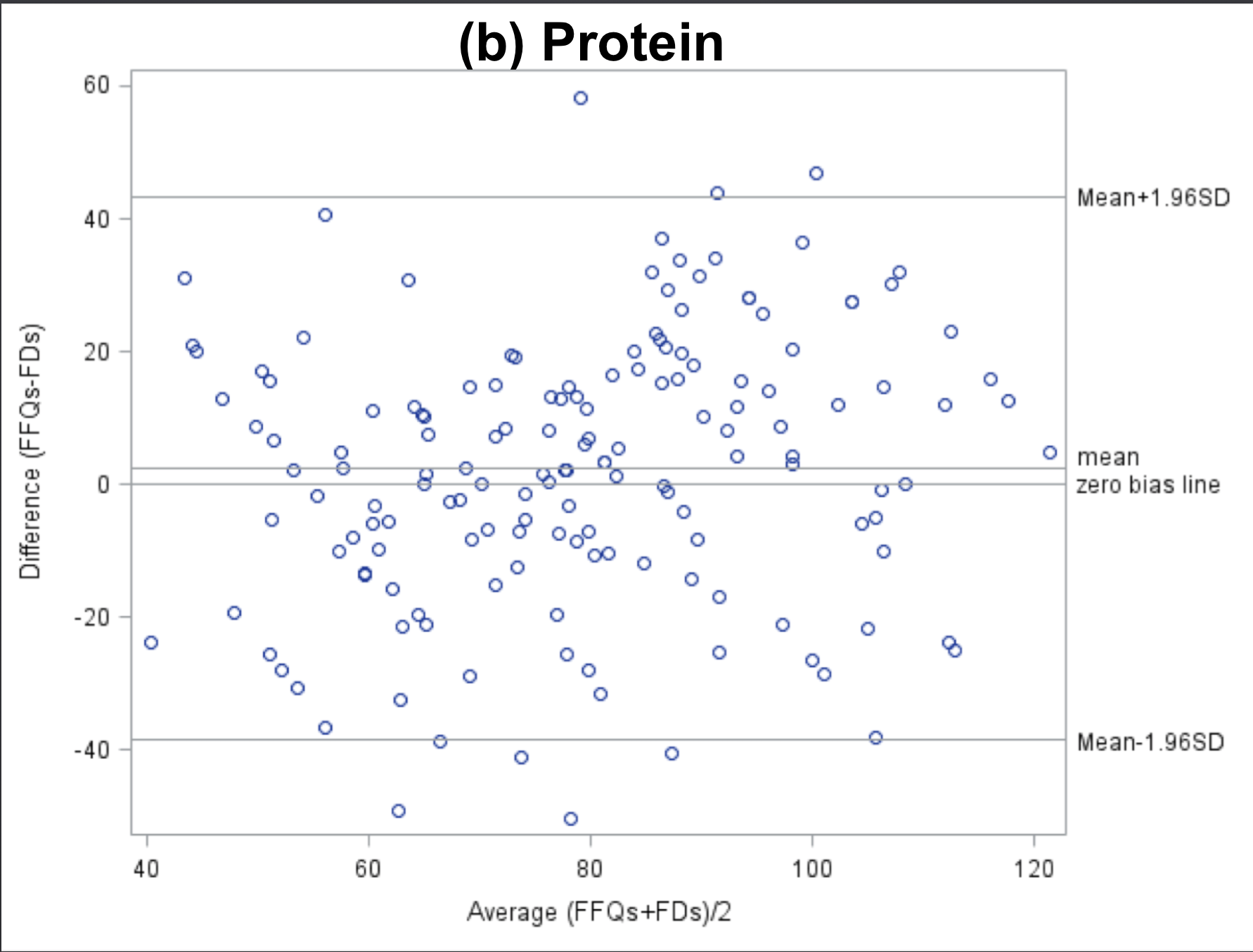 |
| --- | --- |
| (**a**) | (**b**) |
| 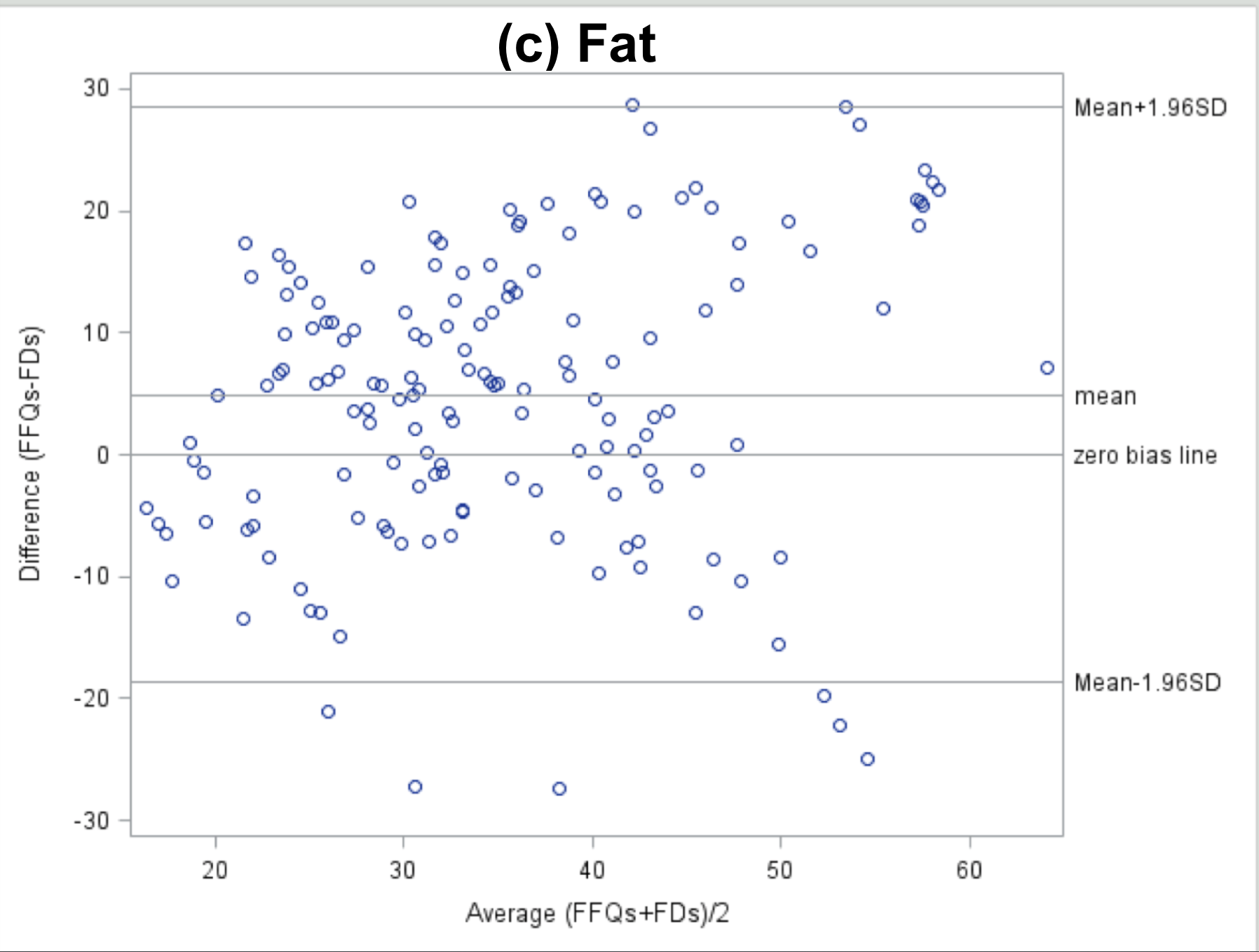 | 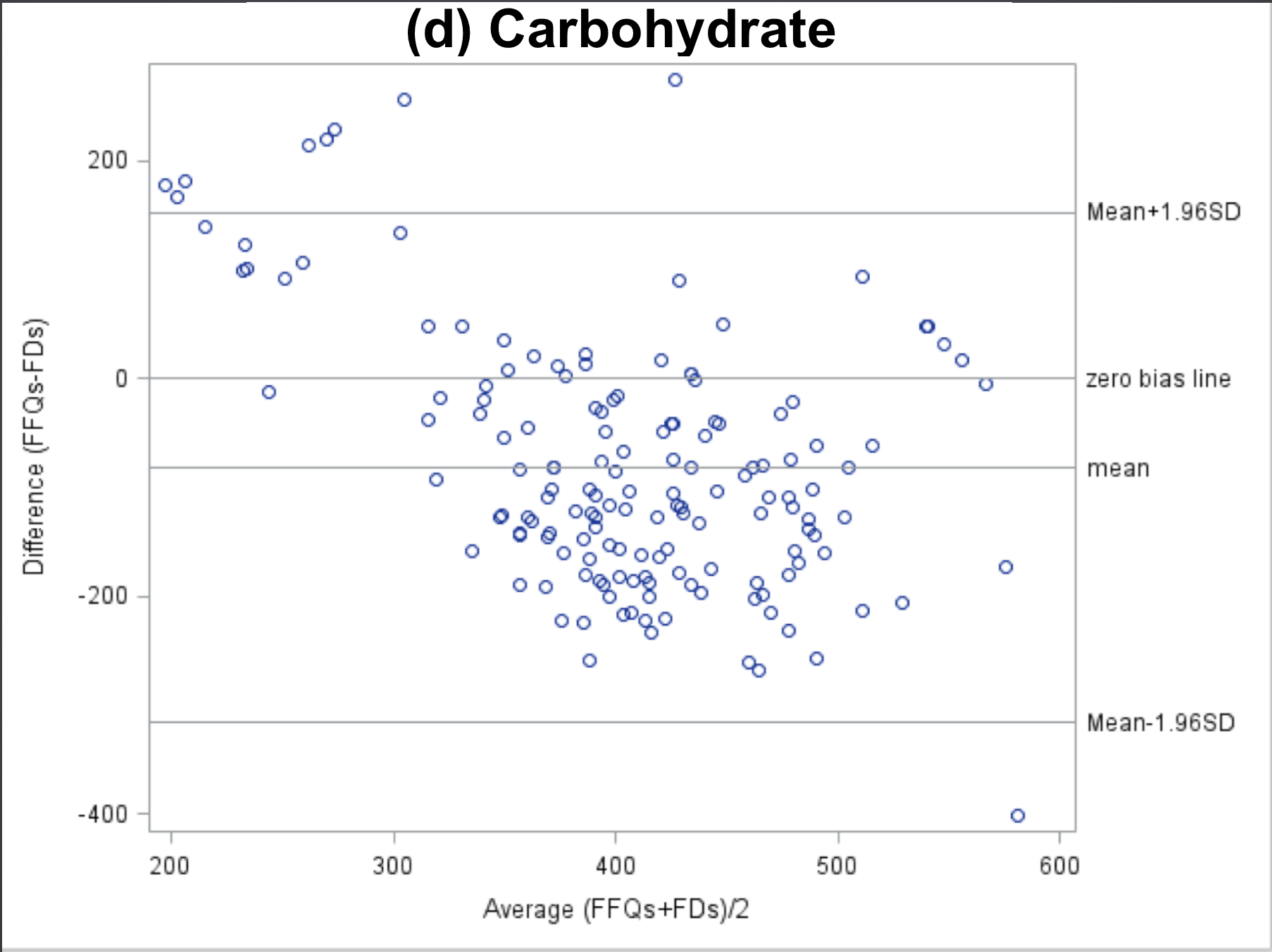 |
| (**c**) | (**d**) |
| 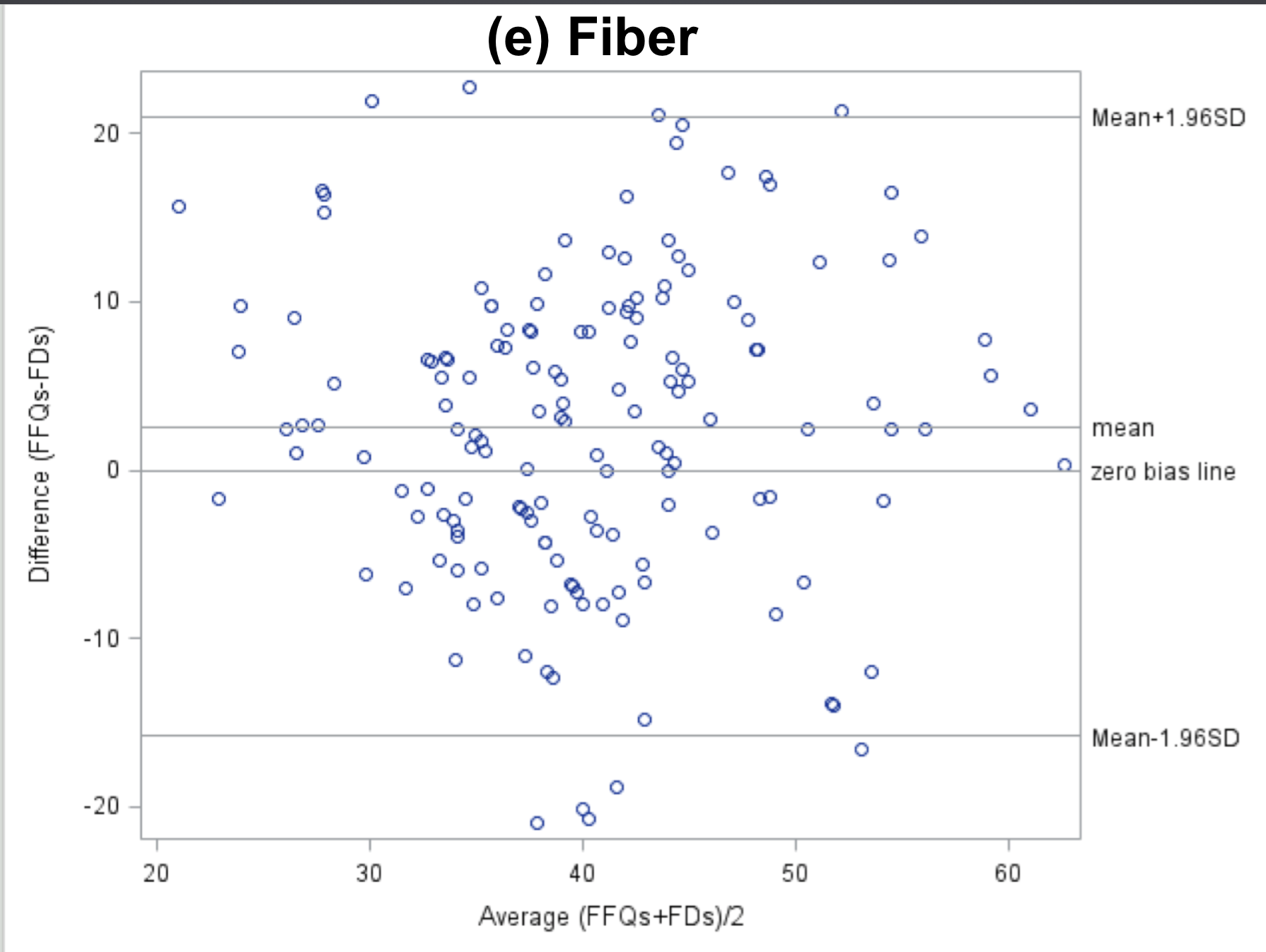 | 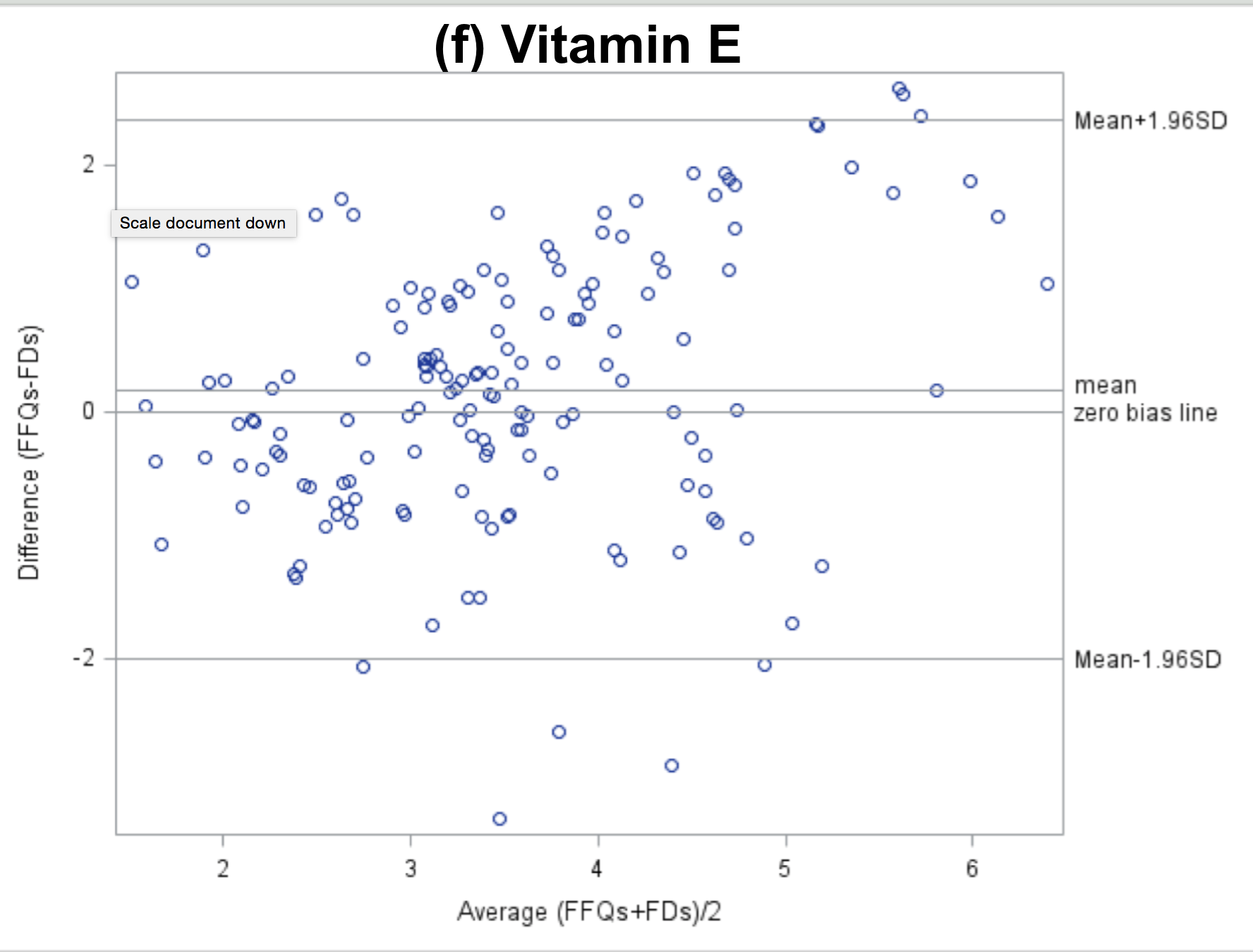 |
| (**e**) | (**f**) |
| 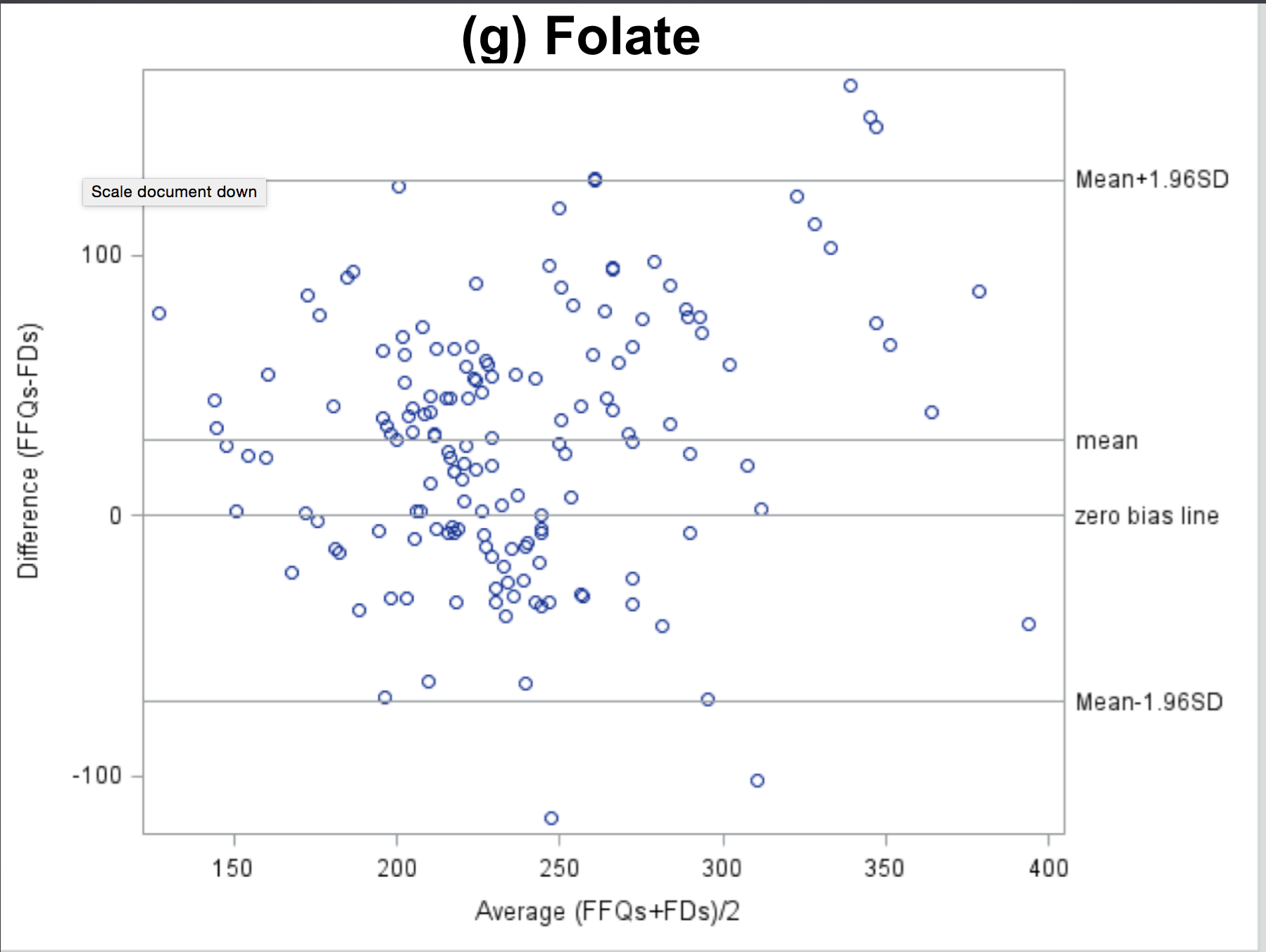 | 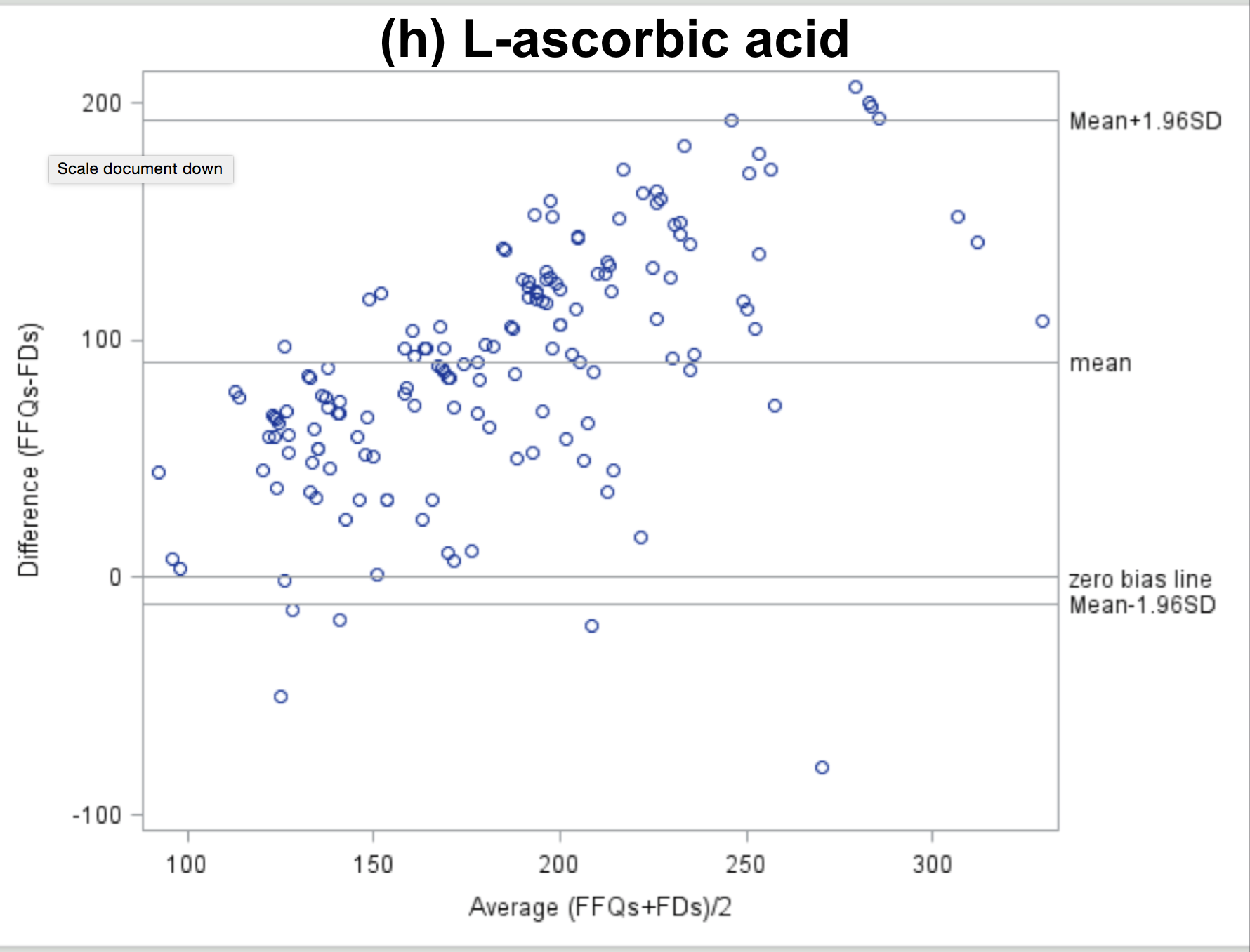 |
| (**g**) | (**h**) |

| 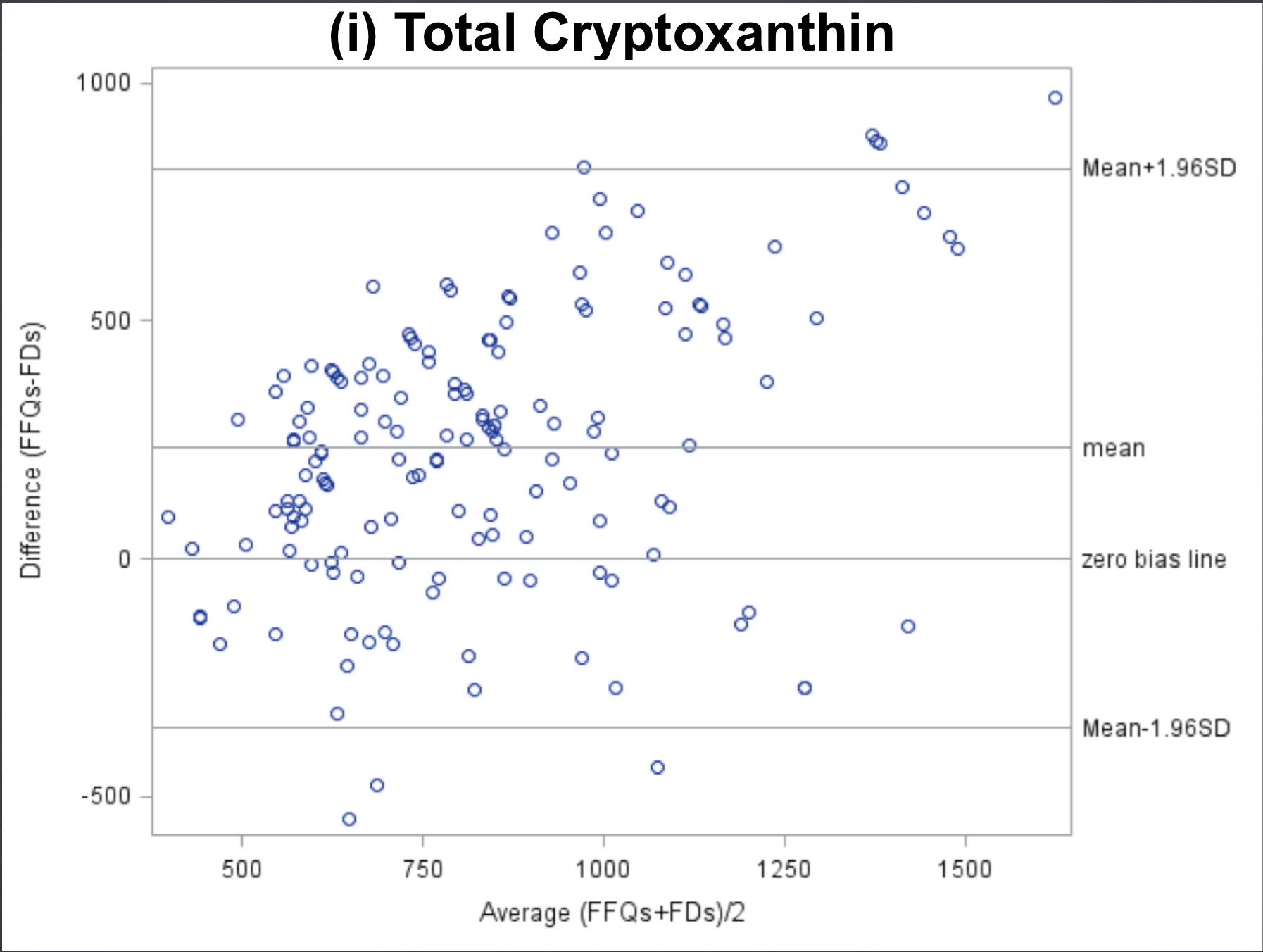 |
| --- |
| (**i**) |

**Figure S2.** Bland–Altman plots for selected nutrient intakes showing agreement between paired means and differences in nutrient intakes measured by Food Frequency Questionnaire (FFQ) and food diary (FD). (**a**) Total energy (kcal/day); (**b**) Protein (g/day); (**c**) fat (g/day); (**d**) carbohydrate available (g/day); (**e**) total dietary fiber (g/day); (**f**) vitamin E (mg/g); (**g**) folate (mcg/day); (**h**) L-ascorbic acid (mg/day); (**i**) total cryptoxanthin (mcg/day).

**Table S1.** Food dishes in each of food groups in the semi-quantitative dish-based Food Frequency Questionnaire (FFQ).

| **Food Group** | **Food Dishes: English Name (Local Name)** |
| --- | --- |
| Grain, Cereal, Bread based | Plain rice (Bhaat, Panta bhaat)  Special rice (Khichuri, Pulao, Biriyani)  Rice cereal (Chira, Muri, Khoi, Murki)  Plain bread (Atta ruti, Pau ruti)  Fried bread (Porota, Luchi)  Home made snacks (Pitha-puli) |
| Vegetable based | Leafy vegetable (Sak)  Mashed vegetable (Bhorta)  Fried vegetable (Bhaji)  Mixed vegetable (Labra)  Vegetable Curry (Torkarir jhole) |
| Legumes, Pulses, Seeds based | Plain dal  Dal with vegetables |
| Fish, Poultry, Meat, Egg based | Fish Fry (Mach bhaji)  Fish curry (Mach er jhole)  Fish curry with vegetable  Fish head with dal or vegetables  Fish egg fry (Maccher dim bhaji)  Dried fish with vegetable  Meat curry with potato  Meat with legumes (Halim)  Meat with, grains, legumes, vegetables (Dhansak)  Meat kabab  Egg curry (Dim er jhole) |
| Milk based | Plain milk (Doodh)  Cottage cheese (chana)  Yogurt (Doi)  Yogurt drink (Ghole, Matha, Borhani)  Thickened milk (Khoa, kheer)  Rice pudding (Payesh)  Vermicelli (Semai)  Sweetmeats (Mishti) |
| Fruits | Fruit  Mashed fruit (Bhorta)  Fruit pickle (Aachar) |
| Beverages | Plain water  Fruit juice  Soft drinks  Tea  Coffee |

**Table S2.** Socio-demographic characteristics of the 47 female heads of household.

|  | ***n*** | **%** |
| --- | --- | --- |
| All participants | 47 |  |
| Sex |  |  |
| Male | 0 | 0.0% |
| Female | 47 | 100.0% |
| Age |  | 0.0% |
| 20–30 | 12 | 25.5% |
| 31–40 | 22 | 46.8% |
| 41–50 | 11 | 23.4% |
| 51–65 | 2 | 4.3% |
| >65 | 0 | 0.0% |
| BMI |  |  |
| <18.5 | 7 | 14.9% |
| 18.5–24.9 | 29 | 61.7% |
| 25.0+ | 11 | 23.4% |
| Education |  |  |
| Illiterate | 6 | 12.8% |
| Able to write | 24 | 51.1% |
| Primary Education | 6 | 12.8% |
| Secondary Education | 9 | 19.1% |
| Higher Secondary Education | 2 | 4.3% |
| Job Type |  |  |
| Factory Labor | 0 | 0.0% |
| Businessman | 1 | 2.1% |
| Housewife | 45 | 95.7% |
| Student | 1 | 2.1% |

BMI: Body mass index.

**Table S3.** Degree of association and level of agreement between average daily food group intakes by FD and FFQ reported by the 47 female heads of households.

| **Food Group (Subgroup)** | **Average Daily Consumption (Serving/Day)** | | | | **Correlation Coefficient between  FD and FFQ** | | |
| --- | --- | --- | --- | --- | --- | --- | --- |
|  | **FD (*n* = 47)** | | **FFQ (*n* = 47)** | | **Unadjusted ^1^** | **Energy- Adjusted ^1^** | **Corrected ^3^** |
|  | **Mean** | **SD** | **Mean** | **SD** | **Pearson** | **Pearson** | **Pearson** |
| Grain, Cereal, Bread based | 4.99 | 1.64 | 8.75 | 2.32 | 0.40 | 0.29 | 0.50 |
| (Rice) | 4.13 | 1.21 | 6.47 | 1.63 | 0.23 | 0.34 | 0.41 |
| (Bread) | 0.77 | 1.54 | 2.25 | 2.38 | 0.45 | 0.62 | 0.72 |
| Vegetable based | 1.14 | 1.78 | 0.56 | 0.29 | 0.15 | 0.15 | 0.36 |
| (Leafy Vegetable) | 0.03 | 0.10 | 0.11 | 0.10 | 0.23 ^≈^ | 0.43 | 0.47 |
| (Other Vegetable) | 1.11 | 1.78 | 0.44 | 0.25 | 0.13 | 0.29 | 0.41 |
| Legumes, Pulses, Seeds based | 0.19 | 0.30 | 0.18 | 0.14 | 0.41 | 0.16 | 0.43 |
| Fish, Poultry, Meat, Egg based | 0.52 | 0.33 | 0.60 | 0.65 | 0.13 | 0.20 | 0.42 |
| (Meat) | 0.05 | 0.13 | 0.10 | 0.20 | 0.61 | 0.25 | 0.64 |
| (Fish) | 0.25 | 0.31 | 0.42 | 0.60 | 0.20 | 0.38 | 0.52 |
| (Eggs) | 0.08 | 0.21 | 0.07 | 0.07 | 0.16 | 0.56 | 0.68 |
| Milk based | 0.03 | 0.14 | 0.68 | 0.65 | 0.47 | 0.64 | 0.68 |
| Fruits | 1.57 | 4.24 | 0.91 | 0.94 | 0.50 | 0.35 | 0.71 |
| Beverages | 0.09 | 0.32 | 1.04 | 2.20 | 0.40 | 0.76 | 0.80 |

^≈^ *p* > 0.1, for all other correlation, *p* < 0.05. ^1^ Calculated based on log-transformed value of average daily consumptions. ^2^ Calculated based on log-transformed value of average daily consumptions with adjustment for total energy intake using the residual method. ^3^ Correction were calculated based on energy-adjusted correlation according for random within-individual error in the two 3-day FD.

**Table S4.** Degree of association and level of agreement between average daily nutrients intakes by FD and by FFQ reported by the 47 female heads of households.

|  | **Average Daily Dietary Intake (Unit/Day)** | | | | | | | **Correlation Coefficient between FD  and FFQ** | | |
| --- | --- | --- | --- | --- | --- | --- | --- | --- | --- | --- |
|  | **FD** | | **FFQ** | | **Difference  (FFQ-FD) ^1^** | | **FD:FFQ** | **Unadjusted ^2^** | **Energy- Adjusted ^3^** | **Corrected ^4^** |
| **Nutrients (Unit)** | **Mean** | **STD** | **Mean** | **STD** | **Mean** | **STD** | **%** | **Pearson *r*** | **Pearson *r*** | **Pearson *r*** |
| Protein (g) | 46.77 | 1.32 | 51.24 | 1.46 | 2.99 | 16.23 | 109.56% | 0.33 | 0.41 | 0.61 |
| Fat (g) | 17.80 | 1.50 | 22.10 | 1.78 | 7.36 | 14.37 | 124.16% | 0.28 | 0.48 | 0.93 |
| Carbohydrate available (g) | 287.34 | 1.87 | 236.46 | 1.29 | −72.74 | 97.48 | 82.29% | 0.33 | 0.39 | 0.70 |
| Total dietary fiber (g) | 24.05 | 1.38 | 26.80 | 1.39 | 2.16 | 9.90 | 111.43% | 0.33 | 0.35 | 0.72 |
| Ash (g) | 9.89 | 1.33 | 10.69 | 1.34 | 1.07 | 3.97 | 108.09% | 0.11 ^∞^ | 0.20 | 0.41 |
| Calcium (mg) | 290.39 | 1.35 | 386.78 | 1.42 | 109.10 | 171.87 | 133.19% | 0.18 | 0.23 | 0.38 |
| Iron (mg) | 19.83 | 1.51 | 19.03 | 1.59 | −0.08 | 8.55 | 95.97% | 0.28 | 0.18 | 0.25 |
| Magnesium (mg) | 322.43 | 1.38 | 351.37 | 1.38 | 24.17 | 112.14 | 108.98% | 0.24 | 0.18 | 0.29 |
| Phosphorus (mg) | 834.69 | 1.45 | 765.01 | 1.38 | −77.30 | 311.32 | 91.65% | 0.14 | 0.15 | 0.23 |
| Potassium (mg) | 1252.93 | 1.33 | 1554.75 | 1.38 | 347.08 | 571.33 | 124.09% | 0.18 | 0.16 | 0.23 |
| Sodium (mg) | 715.22 | 1.95 | 815.95 | 2.10 | 102.69 | 981.81 | 114.08% | 0.19 | 0.21 | 0.41 |
| Zinc (mg) | 9.03 | 1.35 | 8.00 | 1.33 | −0.87 | 2.94 | 88.59% | 0.21 | 0.18 | 0.42 |
| Copper (mg) | 2.66 | 1.19 | 2.92 | 1.23 | 0.30 | 0.73 | 109.77% | 0.13 | 0.15 | 0.23 |
| Vitamin A (mcg) | 290.67 | 1.43 | 184.00 | 1.82 | −103.42 | 201.88 | 63.30% | 0.13 | 0.31 | -^≠^ |
| Retinol (mcg) | 23.81 | 2.33 | 20.75 | 3.33 | 3.18 | 52.79 | 87.15% | 0.22 | 0.21 | 0.39 |
| Beta-carotene equivalents (mcg) | 3173.82 | 1.54 | 2022.65 | 2.52 | −639.16 | 3358.32 | 63.73% | 0.16 | 0.22 | 0.36 |
| Alpha-carotene (mcg) | 544.28 | 1.70 | 680.19 | 2.56 | 284.62 | 762.82 | 124.97% | 0.21 | 0.28 | 0.47 |
| Beta-carotene (mcg) | 2776.81 | 1.46 | 1694.72 | 2.20 | −864.53 | 2083.81 | 61.03% | 0.14 | 0.25 | 0.40 |
| Total Cryptoxanthin (mcg) | 358.42 | 1.66 | 493.50 | 2.19 | 230.04 | 476.18 | 137.69% | 0.09 ^≈^ | 0.22 | 0.39 |
| Vitamin D (mcg) | 1.83 | 1.25 | 2.01 | 1.77 | 0.84 | 1.32 | 109.84% | 0.10 ^≈^ | 0.31 | 0.48 |
| Vitamin E (mg) | 3.16 | 1.22 | 3.15 | 1.45 | 0.23 | 1.40 | 99.68% | 0.13 | 0.15 | 0.25 |
| Thiamin (mg) | 1.61 | 1.11 | 1.63 | 1.13 | 0.03 | 0.24 | 101.24% | 0.21 | 0.12 ^∞^ | 0.19 |
| Riboflavin (mg) | 1.43 | 1.10 | 1.45 | 1.14 | 0.04 | 0.21 | 101.40% | 0.20 | 0.18 | 0.28 |
| Niacin equivalents (mg) | 5.90 | 1.42 | 6.82 | 1.71 | 1.73 | 4.15 | 115.59% | 0.15 | 0.15 | 0.21 |
| Niacin, preformed (mg) | 10.49 | 1.41 | 9.06 | 1.27 | −1.91 | 3.10 | 86.37% | 0.23 | 0.15 | 0.21 |
| Niacin equivalents from tryptophan (mg) | 7.10 | 1.30 | 6.67 | 1.57 | 0.02 | 3.41 | 93.94% | 0.21 | 0.25 | 0.33 |
| Vitamin B6 (mg) | 1.89 | 1.15 | 1.89 | 1.19 | 0.04 | 0.38 | 100.00% | 0.19 | 0.20 | 0.29 |
| Folate (mcg) | 130.06 | 1.34 | 156.82 | 1.40 | 24.96 | 62.05 | 120.58% | 0.23 | 0.24 | 0.34 |
| L-ascorbic acid (mg) | 60.18 | 1.51 | 122.29 | 1.79 | 72.26 | 77.03 | 203.21% | 0.14 | 0.16 | 0.27 |

^≈^ *p* > 0.1, ^∞^ 0.05≤ *p* < 0.1, for all other correlation, *p* < 0.05. ^1^ Difference between intake measured by FFQ and intake measured by FD. ^2^ Correlations were calculated based on log-transformed value of average daily consumptions. ^3^ Correlations were calculated based on log-transformed value of average daily consumptions with adjustment for total energy intake using the residual method. ^4^ Correction were calculated based on energy-adjusted correlation according for random within-individual error in the two 3-day FD. ^≠^ Correction coefficient not calculated due to very large ratio of within-person to between-person variances.
